# Effect of Elderberry (*Sambucus nigra* L.) Extract Intake on Normalizing Testosterone Concentration in Testosterone Deficiency Syndrome Rat Model Through Regulation of 17β-HSD, 5α-Reductase, and CYP19A1 Expression

**DOI:** 10.3390/nu16234169

**Published:** 2024-11-30

**Authors:** Jiyeon Kim, Jinho An, Youngcheon Song, Mincheol Jang, Hyunseok Kong, Sangbum Kim

**Affiliations:** 1 KOSABIO Inc., Namyangju-si 12106, Republic of Korea; shelly7285@kosabio.com (J.K.); romang1230@kosabio.com (J.A.); 2 College of Pharmacy, Sahmyook University, Seoul 01795, Republic of Korea; alexsongsu@syu.ac.kr; 3 Hurum Co., Ltd., Geumcheon-gu, Seoul 08505, Republic of Korea; jmc@hurumcorp.com; 4 Department of Animal Science, Sahmyook University, Seoul 01795, Republic of Korea; 5 PADAM Natural Material Research Institute, Sahmyook University, Seoul 01795, Republic of Korea

**Keywords:** testosterone deficiency syndrome, elderberry, *Sambucus nigra* L., total testosterone, free testosterone, testosterone synthesis

## Abstract

**Background/Objectives.** Men experience Leydig cell and mitochondrial dysfunction due to the accumulation of reactive oxygen species during aging, leading to hormonal imbalances in the body. This results in symptoms of testosterone deficiency syndrome (TDS) as testosterone levels decline. Consequently, there is a growing need for alternative therapies, such as phytotherapy, to regulate testosterone secretion. **Methods.** In this study, we evaluated the potential of elderberry extract powder (KSB191) as a functional ingredient for improving TDS by analyzing its mechanism in regulating testosterone imbalance. The major compounds of KSB191 were rutin and fructose–leucine, and the efficacy of KSB191 was confirmed by observing increases in total testosterone, free testosterone, and sperm motility in an aged rat model with decreased testosterone levels. Additionally, we assessed safety by analyzing levels of prostate-specific antigen, alanine aminotransferase, aspartate aminotransferase, and creatinine. **Results.** To confirm the effectiveness of KSB191 in increasing testosterone synthesis and inhibiting its breakdown, we analyzed the expression levels of genes related to testosterone synthesis and degradation in the testis tissue. KSB191 not only increases the expression levels of enzymes (*3β-HSD*, *CYP17A1*, and *17β-HSD*) that catalyze testosterone synthesis in Leydig cells, but also reduces the expression of enzymes (5α-reductase and *CYP19A1*) that degrade testosterone, thereby enhancing testosterone production in the body. **Conclusions.** KSB191 is predicted to be a novel functional ingredient that acts on Leydig cells and increases testosterone synthesis (particularly, the increase in free testosterone), ultimately alleviating the symptoms of TDS.

## 1. Introduction

Aging is accelerated by a variety of complex factors, including modern lifestyle habits, stress, environmental hormones, and diseases. Accordingly, interest in aging is increasing across various age groups, and aging is recognized as a ‘natural phenomenon’ and ‘disease’ that requires prevention and treatment. The World Health Organization assigned a disease code (XT9T) to aging in the 2018 International Classification of Diseases and research on aging is actively being conducted worldwide [1,2]. Among the various anti-aging treatments, hormones have attracted increasing attention. Beyond the 30s, cell function deteriorates and reactive oxygen species (ROS) cannot be normally removed, leading to excessive accumulation and mitochondrial dysfunction, the center of cellular energy metabolism. This causes a hormonal imbalance in the body [3]. Among several steroid hormones, androgens refer to all male hormones that affect the growth, development, and function of the male reproductive system. Testicular function deteriorates due to excessive accumulation of ROS, as well as various factors, such as disease and stress, which damage mitochondria of Leydig cells. These result in the decreased expression of enzymes related to steroidogenesis. Ultimately, mitochondrial dysfunction in the Leydig cell may be a direct cause of decreased testosterone biosynthesis and secretion [4]. Testosterone, the most representative male hormone, peaks at approximately 30 years of age and gradually decreases by approximately 1–2% every year thereafter. The rate of decline can gradually accelerate as they enter their 40s and 50s [5]. In general, the main symptoms of decreased testosterone levels are clinical and biochemical syndromes accompanied by typical aging symptoms, such as decreased sexual function, erectile dysfunction, weight gain, decreased bone density, decreased muscle mass, muscle weakness, sleep disorders, memory decline, and depression. This condition is called testosterone deficiency syndrome (TDS) [6,7,8,9]. TDS is diagnosed when the total testosterone level is < 350 ng/dL (normal range is 350 ng/dL or more) and associated symptoms are present [10]. The prevalence rate is usually <20%, until reaching the age of 60 years, and increases by approximately 10% every 10 years. In particular, 25–30% of men over 60 years old have decreased testosterone levels. TDS accelerates the symptoms of aging in men and has a substantial impact on physical, mental, and social health, thereby reducing their quality of life [7]. Furthermore, male patients with COVID-19 experienced much higher severity and mortality than those of female patients. This was found to be closely related to low testosterone; therefore, the importance of testosterone to men’s health is being emphasized [11].

Testosterone replacement therapy (TRT) is used to improve TDS symptoms by restoring low testosterone levels in men. Although these treatments have the advantage of improving TDS symptoms, certain risks and possible side effects limit their long-term efficacy. The known side effects include polycythemia, sleep apnea, benign prostatic hyperplasia (BPH), prostate cancer, and increased risk of cardiovascular disease [5,8,12]. TRT does not improve the function of Leydig cells that synthesize testosterone; it only supplements insufficient testosterone [13]. Therefore, alternative therapies with fewer side effects, such as phytotherapy, are needed to improve the function of Leydig cells to continuously regulate testosterone synthesis and secretion in the body and alleviate TDS.

Elderberry (*Sambucus nigra* L.) is a species complex of flowering plants belonging to the Adoxaceae family native to most of Europe. It is also called black elder because it bears abundant black fruit, mainly in the fall. It is a safe material that has been used for a long time as a food ingredient in fruit juice, jam, syrup, jelly, wine, etc. Almost all parts of the fruit, including leaves, stems, and roots, are used [14]. Elderberries contain many ingredients with excellent antioxidant and antiviral properties that can provide many health benefits to the human body, making them effective in preventing colds and strengthening immunity. In Europe, it is used to treat viral infections, fever, cold, and influenza, as well as respiratory, gastrointestinal, and skin diseases [15,16,17].

Recently, as life expectancy and the desire to improve the quality of life in old age has increased, awareness of TDS and research on natural functional compounds to improve symptoms has also rapidly increased [18,19]. Elderberry is rich in active ingredients, such as anthocyanins and polyphenols, and has excellent anti-inflammatory and antioxidant effects [20,21]; however, more research is needed on other useful and unknown properties, and its TDS-related efficacy has not been revealed. Based on previous research, we confirmed that fructose–leucine (FL), a previously unknown active ingredient and a monosaccharide–amino acid, is present in elderberry extract; this plays an antioxidant role by significantly reducing ROS production in TM3 Leydig cells. Elderberry extract and FL are effective in improving testosterone reduction by increasing the levels of enzymes involved in testosterone biosynthesis, which are reduced due to Leydig cell dysfunction caused by ROS [22]. Furthermore, we analyzed the biomarkers of elderberry extract for controlling testosterone imbalance in elderly Sprague–Dawley (SD) rats and evaluated their potential as TDS symptom-improving functional materials to maintain male health.

## 2. Materials and Methods

### 2.1. Preparation of the Extract

Dried elderberry fruits (*Sambucus nigra* L.) were purchased from N BIOTEHC Co., Ltd. (Hwaseong-si, Gyeonggi-do, Republic of Korea), and the country of origin was Poland. Dried elderberries were weighed and purified water of 10 times the amount of elderberry was added to conduct an extraction at 80 °C for 3 h. Subsequently, the extracts were filtered (60 mesh) and concentrated under reduced pressure to 15.0 Brix. The concentrate was mixed with dextrin and freeze dried into powder. The freeze-dried extract powder (elderberry fruits extract and dextrin mixture 7:3, 48.74% yield) was named KSB191 (Figure S1). All processes were performed at a good manufacturing practice (GMP) production facility (Samwoodayeon Inc., Geumsan-gun, Chungcheongnam-do, Republic of Korea; WORLDWAY Co., Ltd., Jeonui-myeon, Sejong-si, Republic of Korea).

### 2.2. Isolation and Purification of KSB191 Components

To isolate and purify the KSB191 component, the compound was fractionated using medium-pressure liquid chromatography (MPLC, TELEDYNE-Isco Torrent CombiFlash, Lincoln, NE, USA), and then, to increase the purity of the compound, preparative high-performance liquid chromatography (prep-HPLC, LC/Forte/R, YMC, Kyoto, Japan) was used to isolate the compounds through additional fractionation. The column was operated using YMC-Pack ODS-A (YMC Co., Ltd.), and water containing acetonitrile (ANC) and 0.05% trifluoroacetic acid was used as the mobile phase; high-purity separation of compounds was performed at a flow rate of 15 or 18 mL/min.

### 2.3. Major Compounds Analysis in KSB191

Rutin in KSB191 was analyzed using standardized HPLC. A rutin standard (PhytoLab GmbH and Co. KG., Vestenbergsgreuth, Germany) was dissolved in 60% methanol and prepared at a concentration of 0.1 mg/mL as standard stock solutions. The standard stock solution was diluted with 60% methanol, prepared at concentrations of 5, 10, 30, 50, and 100 μg/mL, and used as a standard solution for calibration curves. Each solution was then assayed on an HPLC system (1260 Infinity II, Agilent Technologies Inc., Santa Clara, CA, USA) using an ultraviolet detector (355 nm) and the separation was performed on a Gemini C18 110 Å column (4.6 × 250 mm, 5 μm, Phenomenex Inc., Torrance, CA, USA). FL in KSB191 was determined using standardized liquid chromatography–mass spectrometry (LC/MS). FL standards (Santa Cruz Biotechnology Inc., Dallas, TX, USA) were dissolved in 60% methanol and prepared at a concentration of 0.1 mg/mL as standard stock solutions. Each solution was then assayed on an LC/MS system (1290 Infinity II 6495 TripleQuad LC/MS, Agilent Technologies Inc.) and the separation was performed on a Dcpak PTZ column (2.1 × 100 mm, 3.0 μm, Daicel Corporation, Kita-ku, Osaka, Japan).

### 2.4. Animal and Experimental Protocols

Specific pathogen-free SD rats were purchased from Samtako Bio Co., Ltd. (Osan, Gyeonggi-do, Republic of Korea). Male SD rats weighing 650–750 g of 48 weeks old were used as the TDS animal model of aging and housed in plastic cages containing 2–3 rats each. All test animals had ad libitum access to a normal diet and water for 12 weeks after an acclimatization period of one week before starting the experiment. The housing room was maintained at 22 ± 2 °C and a humidity of 50 ± 20% on a 12 h light/dark cycle. All experimental protocols were approved and performed according to the ethical guidelines issued by the Institutional Animal Care and Use Committee of Sahmyook University (approval number: SYUIACUC, 2021–022). Animal clinical symptoms were monitored once a day. The laboratory animals were randomly assigned to four groups of seven rats each; a total of 28 rats were used. Each group was orally administered KSB191 suspended in distilled water (G2,130 mg/kg; G3,195 mg/kg; and G4,260 mg/kg) daily for 12 weeks, with a normal control group (G1, vehicle; distilled water).

### 2.5. Measurement of TDS-Related Indicators in Serum

After fasting, rats were anesthetized with 2% *v*/*v* isoflurane in oxygen. Blood was collected from the jugular vein during the study period and from the abdominal vein at the end of the experiment. Serum was separated from whole blood by centrifugation at 10,000 rpm at 4 °C for 5 min. Enzyme-linked immunosorbent assay (ELISA) was performed to confirm TDS-related indicators in the serum (Table S3). Serum total testosterone (TT) levels were measured using an ELISA kit (ab108666, Abcam, Cambridge, UK). To investigate the correlation between age and testosterone deficiency, TDS-related indicators, such as free testosterone (FT), gonadotropin-releasing hormone (GnRH), luteinizing hormone (LH), and follicle-stimulating hormone (FSH) were measured using an ELISA kit (MBS740121, MBS762089, MBS764675, MBS2502190, MyBioSource, Inc., San Diego, CA, USA). To evaluate safety against testosterone increases, serum prostate-specific antigen (PSA) levels were measured using an ELISA kit (MBS267868, MyBioSource, Inc.). All assays were performed according to the manufacturer’s instructions.

### 2.6. Quantitative Real-Time Polymerase Chain Reaction (qRT-PCR) Analysis in Testis Tissue

qRT-PCR was performed to compare the expression levels of androgen synthesis-related genes in the testis tissue. After the testicular tissue of each experimental animal was collected and homogenized, total RNA was isolated using a total RNA purification kit (305-101, GeneAll Biotechnology Co., Ltd., Songpa-gu, Seoul, Republic of Korea), according to the manufacturer’s instructions. For complementary DNA (cDNA) synthesis, 1 μg of RNA was mixed with the HyperScript™ RT premix (GeneAll Biotechnology Co., Ltd.) and oligo (dT) 20 primer. Glyceraldehyde-3-phosphate dehydrogenase (GAPDH) was used as an internal control to normalize 3β-HSD, CYP17A1, 17β-HSD, 5α-reductase, and CYP19A1 (aromatase) mRNA expression levels. Real-time PCR was performed using a SYBR Green PCR Master Mix (Thermo Fisher Scientific Inc., Waltham, MA, USA), each primer, and synthesized cDNA. The sequences of each primer are shown in Table 1. The results were analyzed using QuantStudio5 Real-Time PCR System Software v. 2.6 (Thermo Fisher Scientific Inc.), with GAPDH as a control for the amplified genes.

### 2.7. Western Blot Analysis in Testis Tissue

The testis tissue was homogenized in RIPA cell lysis buffer (GenDEPOT, Baker, TX, USA) with protease inhibitor cocktail solution (GenDEPOT) and incubated to induce cell lysis. The supernatant was obtained to separate total protein, and total protein was quantified at 30 μg using the Bradford reagent (GenDEPOT). Thereafter, a loading sample was prepared using Bolt™ LDS sample buffer (Thermo Fisher Scientific Inc.), Bolt™ reducing agent (Thermo Fisher Scientific Inc.) was added, and the protein was stabilized. Electrophoresis was performed by loading each sample onto a 4–12% Bolt™ Bis-Tris gel (Thermo Fisher Scientific Inc.) and transfer was performed through an iBlot™ 2 PVDF Regular Stack (Thermo Fisher Scientific Inc.). In the blocking process, the membranes were incubated for 2 h with blocking buffer (5% bovine serum albumin (BSA) in Tris-buffered saline containing 0.05% Tween-20, GenDEPOT) and were subsequently incubated with primary antibodies (Santa Cruz Biotechnology Inc.) against 3β-HSD, 17β-HSD, and GAPDH at 4 °C overnight. After repeated washings, the membranes were further incubated with an appropriate dilution of secondary antibody (Abcam) at room temperature for 1 h. Proteins were visualized by West-Q chemiluminescent substrate kit (GenDEPOT) and data were analyzed using the iBright™ FL1500 Imaging System (Thermo Fisher Scientific Inc.).

### 2.8. Sperm Motility Analysis

Epididymis tissues were transferred to a culture dish (Corning Inc., Corning, NY, USA) containing phosphate-buffered saline (PBS, WELGENE Inc., Gyeongsan-si, Gyeongsangbuk-do, Republic of Korea) maintained at 37 °C. After liquefied sperm were collected and diluted in PBS (1:19), the sample was transferred to a 100-micron standard count chamber slide (Leja Products B.V., GN, Nieuw-Vennep, Netherlands), and sperm motility was analyzed using a microscope (Carl Zeiss AG, Oberkochen, Germany) and a computer-assisted sperm analysis system (Hamilton Thorne Inc., Beverly, MA, USA).

### 2.9. Prostatic Hyperplasia Markers Analysis

Body weight was measured before sacrifice using an electronic scale (OHAUS Corporation, Parsippany, NJ, USA), and prostate tissue was weighed after sacrifice using an electronic scale (CAS Corporation, Yangju, Gyeonggi-do, Republic of Korea). Body weight was measured once a week, and only the body weight before sacrifice is shown in Table 2. Prostate index was calculated as prostate weight/body weight × 100 [23]. The lengths of the long and short axes of the prostate were measured using a digital caliper, and the prostate volume was calculated as 1/2 (a × “b”2), where (a) and (b) are the long and short axes, respectively [24].

### 2.10. Biochemical Analysis in Serum

For biochemical analysis, the blood of experimental animals was collected through the abdominal vein after 12 weeks of administration and centrifuged at 10,000 rpm for 5 min to obtain serum. Safety assessment-related indicators such as aspartate aminotransferase (AST), alanine aminotransferase (ALT), and creatinine (CRE) were measured using a biochemical analyzer (AU480, Beckman Coulter, Inc., Brea, CA, USA).

### 2.11. Statistical Analysis

Statistical analyses were performed using GraphPad Prism^®^ version 5.0 (GraphPad Software Inc., Boston, MA, USA). The significance of differences between groups was analyzed by one-way analysis of variance (ANOVA) and Dunnett’s multiple comparison tests. A comparison of the old control group (G1) was performed using the *t*-test and Mann–Whitney test for each group. All data are presented as the mean ± standard error of the mean (SEM), and the values were considered statistically significant at *p* < 0.05.

## 3. Results

### 3.1. Compounds Isolated from KSB191

**Figure 1:**
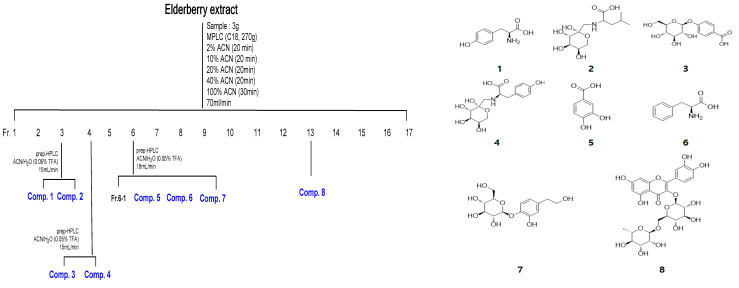
Structures of compounds (**1** to **8**) isolated from KSB191: tyrosine (**1**), *N*-(1-deoxy-D-fructose-1-yl)-L-leucine (**2**), 4-(β-D-glucosyloxy) benzoic acid (**3**), *N*-(1-deoxy-D-fructose-1-yl)-L-tyrosine (**4**), protocatechuic acid (**5**), L-phenylalanine (**6**), 2-hydroxy-4-(2-hydroxyethyl)pheny1β-D-glucopyranoside (**7**), and rutin (**8**).

To identify the components contained in KSB191, the extract powder was repeatedly subjected to MPLC and prep-HPLC to isolate and purify the compounds. Eight compounds were separated, purified, and their structures were identified. The respective structures of the compounds were identified as tyrosine, *N*-(1-Deoxy-D-fructos-1-yl)-L-leucine, 4-(β-D-glucosyloxy) benzoic acid, *N*-(1-Deoxy-D-fructos-1-yl)-L-tyrosine, protocatechuic acid, L-Phenylalanine, 2-Hydroxy-4-(2-hydroxyethyl)phenylβ-D-glucopyranoside, and rutin (Figure 1).

**Figure 2:**
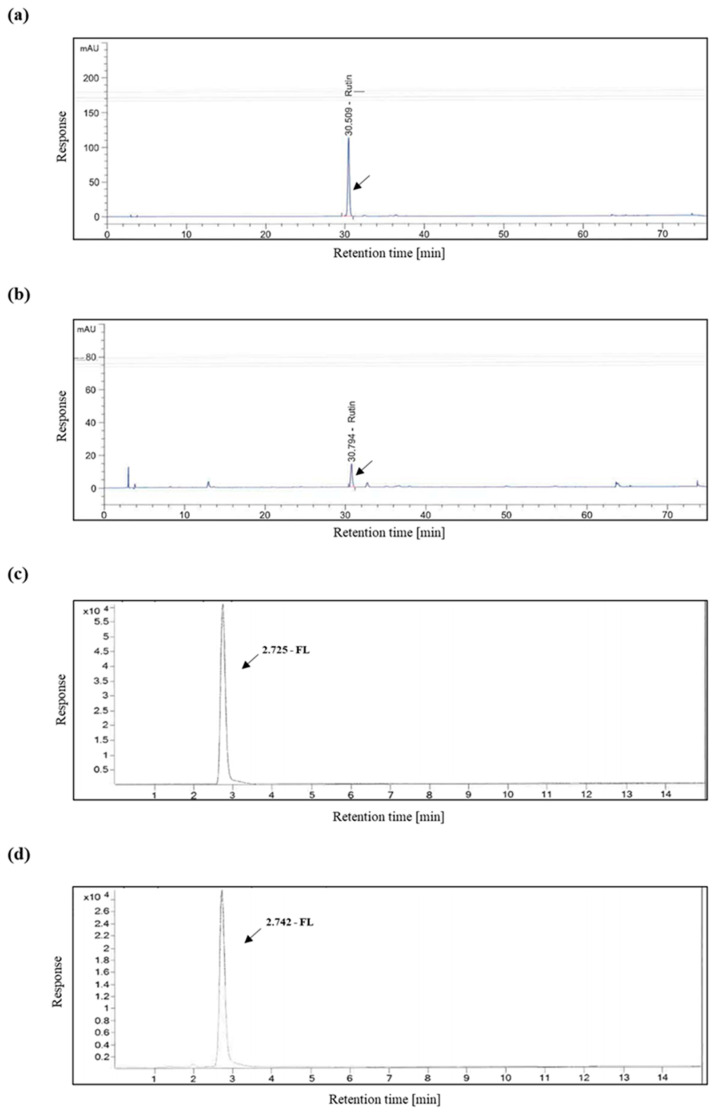
Analysis of content from KSB191. Chromatogram of the standard (**a**,**c**) and KSB191 (**b**,**d**). The content of ingredients in KSB191 is shown using rutin (**b**) and fructose–leucine (**d**) as standards.

KSB191 and rutin standards were analyzed under the same HPLC analysis conditions three times. As a result of HPLC analysis, the average content of the rutin from KSB191 was 2.83 mg/g (Figure 2a,b). The KSB191 and FL standards were analyzed under the same LC/MS conditions three times. The average content of the FL from KSB191 was 5.09 mg/g (Figure 2c,d).

### 3.2. Effect of KSB191 on Hormone Indicators in Serum

**Figure 3:**
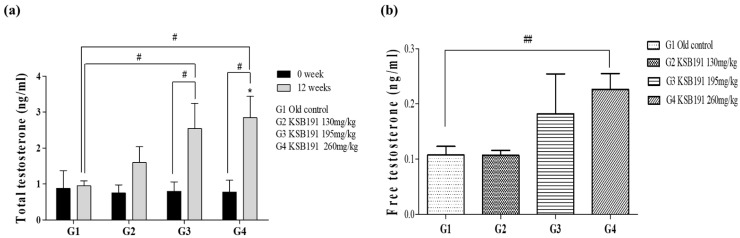
Comparative analysis of the testosterone hormone levels before and after KSB191 administration for 12 weeks. (**a**) Total testosterone and (**b**) Free testosterone levels in serum were analyzed using ELSA. Values are presented as mean ± SEM (*n* = 7). ^#^ *p* < 0.05, ^##^ *p* < 0.01 compared with 0 weeks or G1. Statistical analyses were performed using *t*-test * *p* < 0.05 compared with G1. Statistical analyses were performed using ANOVA. G1, normal control; G2, 130 mg/kg KSB191; G3, 195 mg/kg KSB191; G4, 260 mg/kg KSB191.

Before KSB191 administration, testosterone levels were measured in the 8-week-old and 49-week-old groups to confirm changes in testosterone levels with age (Figure S2). This study was conducted after establishing an aging male animal model and confirming significant differences in testosterone levels. Before administration (0 week), the TT level in the serum of all animals was 0.798 ± 0.163 ng/mL and was separated after ensuring that there was no significant difference between groups. After administration for 12 weeks, TT was analyzed and compared with before administration; there was no significant change in the G1 at 0.948 ± 0.138 ng/mL, but after administration of KSB191 at different concentrations, it increased to 1.596 ± 0.439 ng/mL, 2.540 ± 0.702 ng/mL, and 2.842 ± 0.595 ng/mL, in G2, G3, and G4, respectively. G3 and G4 groups showed a significant increase in TT compared with that before administration. A concentration-dependent increasing trend was confirmed after KSB191 administration compared with that in the G1 group, and there was a significant increase in G3 and G4 (Figure 3a). As a result of analysis of FT levels, which is biologically active testosterone among TT, there was a significant increase at 260 mg/kg (G4, 0.226 ± 0.029 ng/mL) of KSB191 compared with that in the old control (G1, 0.108 ± 0.016 ng/mL). We observed an increasing tendency in a concentration-dependent manner. (Figure 3b).

**Figure 4:**
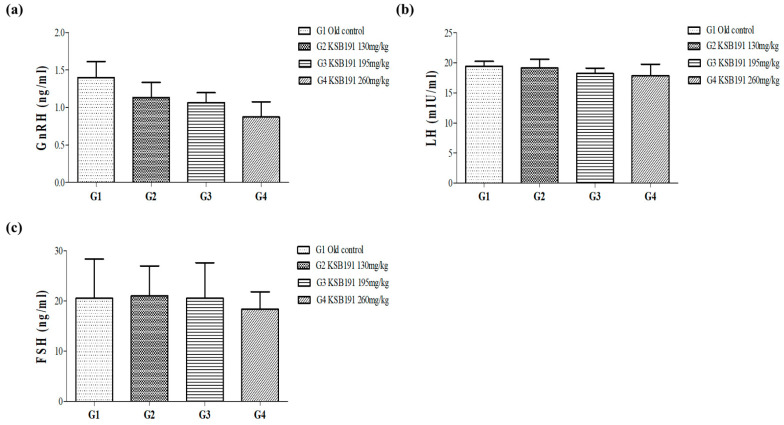
Comparative analysis of the hormone regulation factors by endocrine system after KSB191 administration for 12 weeks. (**a**) gonadotropin-releasing hormone (GnRH), (**b**) leuteinizing hormone (LH) and (**c**) follicle-stimulating hormone (FSH) levels in serum were analyzed using ELISA. Values are presented as mean ± SEM (*n* = 7). Statistical analyses were performed using *t*-test and ANOVA. G1, normal control; G2, 130 mg/kg KSB191; G3, 195 mg/kg KSB191; G4, 260 mg/kg KSB191.

To assess the hormonal regulation of the endocrine system, the serum levels of important hormones along the hypothalamic-pituitary-gonadal axis were analyzed, including GnRH from the hypothalamus and gonadotropins (LH and FSH) secreted from the pituitary gland. No significant differences were found in the serum levels of these hormones between the G1 and G2–4 groups (Figure 4).

### 3.3. Regulation of Testosterone Synthesis Pathway by KSB191

**Figure 5:**
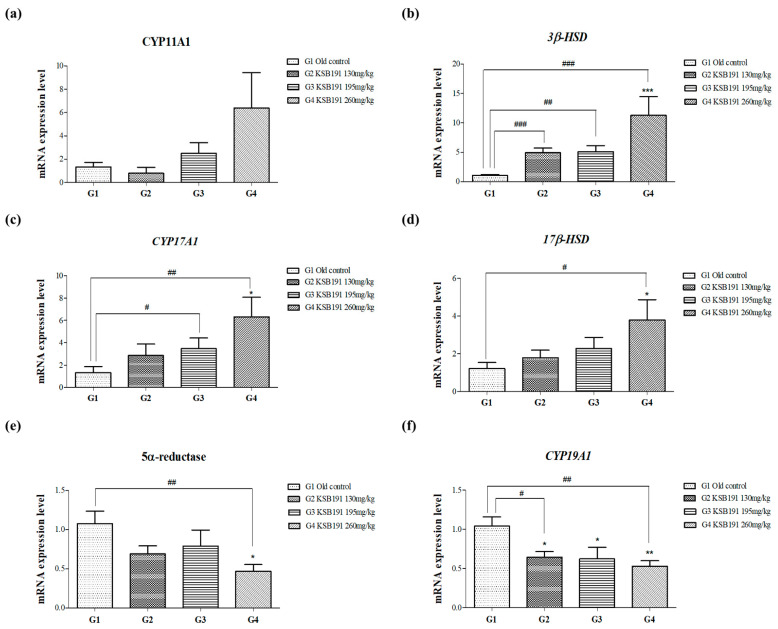
Effects of KSB191 on the mRNA expression levels of steroidogenic enzyme involved in testosterone synthesis. mRNA expression level of steroidogenic gene related to testosterone synthesis ((**a**) *CYP11A1*, (**b**) *3β-HSD*, (**c**) *CYP17A1* and (**d**) *17β-HSD*) and testosterone degradation ((**e**) 5α-reductase and (**f**) *CYP19A1*) were measured in testis tissue. Values are presented as mean ± SEM (*n* = 7). ^#^ *p* < 0.05, ^##^ *p* < 0.01, ^###^ *p* < 0.001 compared with G1. Statistical analyses were performed using *t*-test. * *p* < 0.05, ** *p* < 0.01, *** *p* < 0.001 compared with G1. Statistical analyses were performed using ANOVA. G1, normal control; G2, 130 mg/kg KSB191; G3, 195 mg/kg KSB191; G4, 260 mg/kg KSB191.

**Figure 6:**
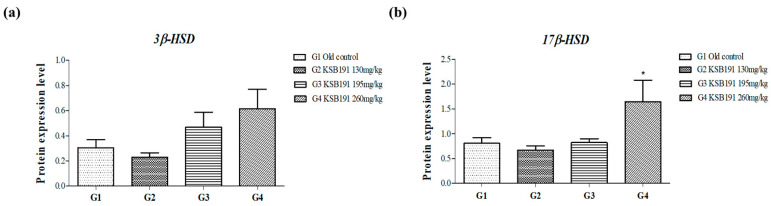
Effects of KSB191 on the protein expression levels of the testosterone synthesis-related genes ((**a**) *3β-HSD* and (**b**) *17β-HSD*) in testis tissue. Values are presented as mean ± SEM (*n* = 7). * *p* < 0.05 compared with G1. Statistical analyses were performed using ANOVA. G1, normal control; G2, 130 mg/kg KSB191; G3, 195 mg/kg KSB191; G4, 260 mg/kg KSB191.

The enzymes of the steroidogenic pathway within the mitochondria of Leydig cells, such as CYP11A1, 3β-HSD, CYP17A1, and 17β-HSD, catalyze testosterone synthesis. In contrast, during testosterone metabolism, 5α-reductase converts testosterone into dihydrotestosterone (DHT), which decreases testosterone levels in the body and contributes to BPH [25]. Additionally, CYP19A1 converts testosterone to estradiol, further reducing the total testosterone concentration. Accordingly, to confirm the effectiveness of KSB191 in increasing testosterone synthesis and inhibiting its breakdown, the mRNA expression levels of genes related to testosterone synthesis in the testis tissue were analyzed. Testosterone synthesis enzymes were compared with the old control (G1) and increased in a concentration-dependent manner with KSB191 (G2–4). The increase was most significant in the G4 group (Figure 5b–d). However, the expression of 5α-reductase and CYP19A1 was decreased by KSB191 (G2–4) compared with those in the G1 group. The expression of 5α-reductase and CYP19A1 was most significantly decreased in the G4 group (Figure 5e,f) compared with the other groups. Based on the mRNA expression results, further proven through protein expression analysis of 3β-HSD and 17β-HSD, the main enzymes involved in testosterone synthesis from beginning to end, a similar trend was observed (Figure 6). Therefore, we confirmed that KSB191 increased low testosterone levels in aging animal models by increasing enzymes related to testosterone synthesis and decreasing enzymes related to testosterone degradation.

### 3.4. Effect of KSB191 on Sperm Motility

**Figure 7:**
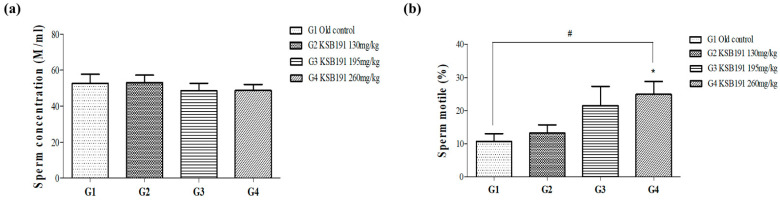
Comparative analysis of the sperm concentration and motility after KSB191 administration for 12 weeks. (**a**) Sperm concentration and (**b**) sperm motile in epididymis tissues. Values are presented as mean ± SEM (*n* = 7). ^#^ *p* < 0.05 compared with G1. Statistical analyses were performed using *t*-test. * *p* < 0.05 compared with G1. Statistical analyses were performed using ANOVA. G1, normal control; G2, 130 mg/kg KSB191; G3, 195 mg/kg KSB191; G4, 260 mg/kg KSB191.

Testosterone has an important effect on fertility; therefore, to confirm the efficacy of KSB191, sperms from the epididymis were collected and analyzed. There was no change in sperm count due to KSB191 treatment compared with that in the G1 group. Sperm motility was confirmed to increase in a concentration-dependent manner by 13.2 ± 2.5%, 21.5 ± 5.8%, and 24.9 ± 3.9% in G2–G4 groups compared with that in the G1 group (10.7 ± 2.3%). There was a significant increase in the G4 group (Figure 7).

### 3.5. Safety of KSB191 on Prostatic Hyperplasia and Liver or Kidney Function

When measuring the body weight of each individual once a week during the 12-week administration period of KSB191, there was no change in body weight before or after the administration of KSB191 compared with that in the G1 group (Figure S3). Organ weights (thymus, kidney, liver, testis, and epididymis) were also not different between G2, G3, and G4 groups (Table S1). Safety against prostatic hyperplasia, which may occur due to increased testosterone, was confirmed, and there was no significant difference in prostate weight, prostate index, or prostate volume after 12 weeks of KSB191 compared with those in the G1 group. Additionally, there was a tendency for PSA to decrease in a concentration-dependent manner due to KSB191, with a significant decrease in the G4 group, compared with that of the G1 group (Table 2). To confirm the safety related to liver and kidney function, serum AST, ALT, and CRE levels were analyzed after administration of KSB191 for 12 weeks. There was no significant difference in G2–4 compared with those in G1, indicating that KSB191 does not negatively affect liver and kidney function. (Table 3).

## 4. Discussion

The mechanism of decreased testosterone levels in males is a mitochondrial dysfunction within Leydig cells in proportion to oxidative stress and is one of the main causes of TDS [26]. We confirmed the efficacy of KSB191 and FL, a major component of KSB191, in increasing testosterone synthesis by suppressing ROS production through previous in vitro studies. These results suggest that KSB191 and FL act as antioxidants, contributing to Leydig cell protection by improving mitochondrial dysfunction and testosterone biosynthesis [22]. In this study, the effect of KSB191 administration on blood testosterone levels was confirmed, and the main mechanism of efficacy was confirmed through analysis of enzymes related to testosterone synthesis and decomposition.

Testosterone is formed through the action of various enzymes in Leydig cells. As a precursor of testosterone, cholesterol is converted to pregnenolone (a precursor of progesterone) and pregnenolone moves to the endoplasmic reticulum, where testosterone is synthesized by enzymes, such as 3β-HSD, CYP17A1, and 17β-HSD [27,28,29]. KSB191 appears to increase the expression of 3β-HSD, thereby promoting the conversion of progesterone. Additionally, the significant increase in CYP17A1 and 17β-HSD expression enhances the synthesis of testosterone from progesterone, suggesting an increase in reduced testosterone levels [30]. It was confirmed that KSB191 confirmed the possibility of increasing testosterone levels in the body by these enzymes. During testosterone metabolism, 5α-reductase converts testosterone into dihydrotestosterone (DHT), which reduces testosterone in the body and causes BPH [25]. Meanwhile, CYP19A1 (aromatase) converts testosterone and androstenedione to estradiol, thereby reducing the overall testosterone concentration [30,31]. The expression of 5α-reductase and CYP19A1 is decreased significantly in KSB191 administration groups. In particular, the effect of reducing the expression of CYP19A1 by KSB191 may be expanded to apply to phytomedicine for lung diseases such as COVID-19 in men [31]. KSB191 acts to enhance testosterone production in the body by inhibiting the breakdown of testosterone and increasing its synthesis As a result of TT analysis, the G2–G4 group increased by approximately 113.4%, 219.3%, and 267.1% compared with G1, respectively, and the increase was statistically significant in the G3 and G4 groups. In particular, it was confirmed that the total testosterone level in the high-dose administration group recovered to a level similar to that of young rats (Figure S2). Testosterone naturally facilitates muscle mass gain and strengthens muscles [32]. The relationship between testosterone and muscle mass increase will be further confirmed in our ongoing studies. Approximately 60–80% of testosterone circulates in the body and tightly binds to sex hormone binding globulin (SHBG), remaining inactive. In contrast, FT (~2%), which is not bound to SHBG, exhibits hormonal activity. Therefore, the concentration of FT in blood plays an important role in testosterone deficiency [33,34]. It was confirmed that FT increased after KSB191 administration, especially in the G3 and G4 groups compared with the G1 group. Questionnaires covering the three areas (psychogenic, somatic, and sexual) of the Aging Males’ Symptoms (AMS) scale have been developed as potential screening tools for males. Bioavailable testosterone such as FT have been shown to correlate significantly with several individual questions of the AMS in multiple studies [35,36,37,38]. The results of increased FT by KSB191 may be more relevant to human psychological, somatic, and sexual effects than increased TT. Therefore, an increase in FT may have a greater effect on improving TDS than an increase in TT.

Testosterone, secreted by Leydig cells in the testes, is a crucial hormone for male reproductive function [39,40]. Based on the results of increased testosterone through the administration of KSB191, the decline in testicular function due to aging was improved, and its effect on the motility of sperm was confirmed. Sperm motility increased with KSB191 administration at different concentrations. In particular, the 260 mg/kg (G4) administration group showed a statistically significant increase of approximately 14.2% compared with the old control (G1). These results show that administration of KSB191 has a positive effect on sperm motility, which is thought to result from increased activity of sperm cells by regulating the microenvironment within the testis by promoting testosterone synthesis and secretion.

Testosterone production is regulated by a feedback loop involving the hypothalamus and pituitary gland. The hypothalamus, a small part of the brain, releases GnRH, which signals the pituitary gland to secrete LH and FSH [40]. Increased testosterone levels in the body ultimately exert negative feedback on the hypothalamus and pituitary gland, reducing the release of GnRH, LH, and FSH. This feedback loop ensures that testosterone levels remain within the normal range. Any disruption in this feedback system can lead to hormonal imbalances and endocrine problems [34,40,41,42]. In this study, no significant changes in the levels of these hormones between the G1 and G2–4 groups was observed. Testosterone bound to SHBG has an increased molecular weight, preventing it from passing through cell membranes, thereby losing its hormonal activity. Most of TT is bound to these proteins [33,34]. SHBG levels tended to increase by KSB191 (*p* > 0.05). The secretion of male hormones increases through normalization of mitochondria that have dysfunction in Leydig cells, but the increase does not exceed the normal level. This appears to be a result of balancing the free testosterone increased by KSB191, and maintaining this, and is not thought to cause the side effects of hormonal imbalance in the body. Some studies have suggested that increasing testosterone levels with TRT may increase prostate size and worsen prostate cancer [43,44]. PSA is a proteolytic enzyme synthesized only in prostate epithelial cells and is mainly used as a tumor marker in screening for prostate cancer; however, it can also increase in chronic prostatitis, prostatic hyperplasia, and prostate calcification [45,46]. In this study, there was no significant change in prostate weight and volume after the administration of KSB191 compared with that in the G1 group. The PSA level tended to decrease in a concentration-dependent manner and showed a significant decrease in the G4 group. Based on these results, it is believed that KSB191 does not cause side effects that may occur due to increased testosterone levels in the prostate.

AST, ALT, and CRE are widely used indicators of liver and kidney damage in clinical practice [47]. There is a close association between reduced testosterone levels and blood lipid abnormalities, including increased cholesterol and TGs [48,49]. In this study, KSB191 administration showed no significant difference compared with the control group, suggesting that it did not cause negative effects, such as toxicity or changes in lipid markers (Table S2).

## 5. Conclusions

In this study, KSB191 has been shown to act on enzymes that increase testosterone concentration, as well as enzymes, such as 5α-reductase and CYP19A1, that reduce testosterone in the body, resulting in an overall higher testosterone concentration. Previous studies have confirmed the efficacy of KSB191 in increasing testosterone synthesis using a mouse Leydig TM3 cell line [22], demonstrating that KSB191 effectively increases testosterone blood concentration and biosynthesis in Leydig cells. Additionally, FL increases testosterone synthesis in Leydig cells, suggesting that FL, a phytochemical of KSB191, has a greater influence on the concentration of testosterone in the blood [22]. Based on the ongoing results, the relationship between Sertoli cell function and testosterone synthesis, mitochondrial function, and ROS will be further investigated in future studies. These results suggest that KSB191 plays a critical role in maintaining mitochondrial function in Leydig cells, protecting cells from various types of damage, ultimately alleviating symptoms associated with TDS. Consequently, KSB191 is a potential novel functional material that can treat TDS, improve men’s health, and restore masculinity through age-defying vitality and youthfulness.

## Figures and Tables

**Table 1:** Primer sequences for qRT-PCR.

Target Gene	Primer Sequence 5′>3′	
	Forward	Reverse
*3β-* *HSD*	AGAACGGCCACGAAGAAGAG	TGGGTCTTAACGCACAAGTGT
*CYP17A1*	CTCTGGGCACTGCATCAC	CAAGTAACTCTGCGTGGGT
*17* *β-* *HSD*	TGGGATCATGCCTAATCCACA	CCAGTTCCCGAATCAGGATAAAA
5α-reductase (*SRD5A2*)	CGGTTTAGCTTGGGTGTCTTC	CCGAGGAAATTGGCTCCAGAA
*CYP19A1*	AACCCCATGCAGTATAATGTCAC	AGGACCTGGTATTGAAGACGAG
*GAPDH*	CAACTTTGGCATTGTGGAAGG	ATGGAAATTGTGAGGGAGATGC

**Table 2:** Safety of KSB191 on prostatic hyperplasia.

		KSB191		
	G1, Old Control	G2, 130 mg/kg	G3, 195 mg/kg	G4, 260 mg/kg
Body weight (g)	712.5 ± 20.0	713.0 ± 17.1	646.2 ± 41.7	724.7 ± 17.4
Prostate Weight (g)	1.558 ± 0.066	1.512 ± 0.173	1.410 ± 0.110	1.513 ± 0.122
Prostate Index (%)	0.22 ± 0.01	0.21 ± 0.02	0.23 ± 0.03	0.21 ± 0.02
Prostate Volume (mm^3^)	10,411.03 ± 697.69	8725.03 ± 654.32	10,584.27 ± 889.43	9353.07 ± 422.38
PSA (pg/mL)	19.802 ± 0.993	17.316 ± 1.173	15.354 ± 1.801	14.460 ± 0.855 ^##,^*

Prostate weight and volume (mm^3^) were measured, and the prostate index was calculated [prostate weight (g)/body weight (g) × 10]. Values are presented as mean ± SEM (*n* = 7). ^##^ *p* < 0.01 compared with G1. Statistical analyses were performed using *t*-test. * *p* < 0.05 compared with G1. Statistical analyses were performed using ANOVA.

**Table 3:** Safety of KSB191 on liver and kidney functions.

		KSB191		
	G1, Old Control	G2, 130 mg/kg	G3, 195 mg/kg	G4, 260 mg/kg
AST (U/L)	113.3 ± 10.4	87.0 ± 6.9	100.4 ± 9.8	85.9 ± 6.5
ALT (U/L)	47.4 ± 3.4	43.7 ± 2.7	44.6 ± 1.8	39.3 ± 1.6
CRE (mg/dL)	0.5 ± 0.08	0.3 ± 0.03	0.4 ± 0.13	0.4 ± 0.08

AST, aspartate aminotransferase; ALT, alanine aminotransferase; CRE, creatine. Values are presented as mean ± SEM (*n* = 7). Statistical analyses were performed using *t*-test and ANOVA.

## Data Availability

The datasets used and/or analyzed during the current study are available from the corresponding author on reasonable request.

## Supplementary Materials

The following supporting information can be downloaded at: https://www.mdpi.com/article/10.3390/nu16234169/s1, Figure S1: Extraction method of KSB191; Figure S2: Comparative analysis of the total testosterone levels in young and old controls before KSB191 administration; Figure S3: Comparative analysis of body weight before and after KSB191 administration for 12 weeks; Table S1: Safety of KSB191 on organ weights; Table S2: Effect of KSB191 on lipid metabolism-related indicators; Table S3: The sensitivity and the inter and intra variations of the ELISA kits.
