# Supplementary material for: Effect of Elderberry (Sambucus nigra L.) Extract Intake on Normalizing Testosterone Concentration in Testosterone Deficiency Syndrome Rat Model Through Regulation of 17β-HSD, 5α-Reductase, and CYP19A1 Expression
Source: Nutrients. 2024 Nov 30;16(23):4169. doi: 10.3390/nu16234169 (PMC11644235; doi:10.3390/nu16234169)
Supplement: Supplementary file 1 [file nutrients-16-04169-s001.zip › nutrients-3296492-supplementary.pdf]

Supplementary Materials

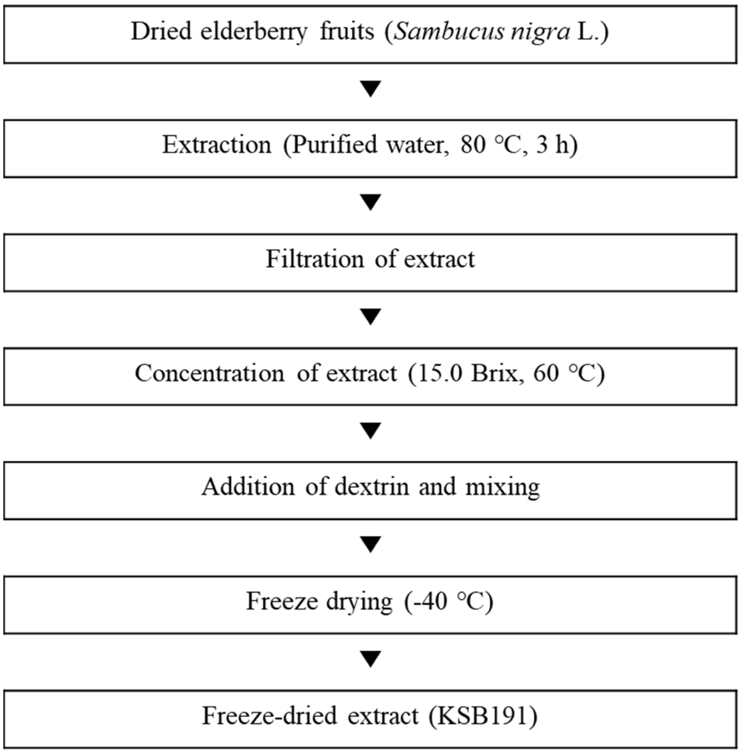

Figure S1. Extraction method of KSB191.

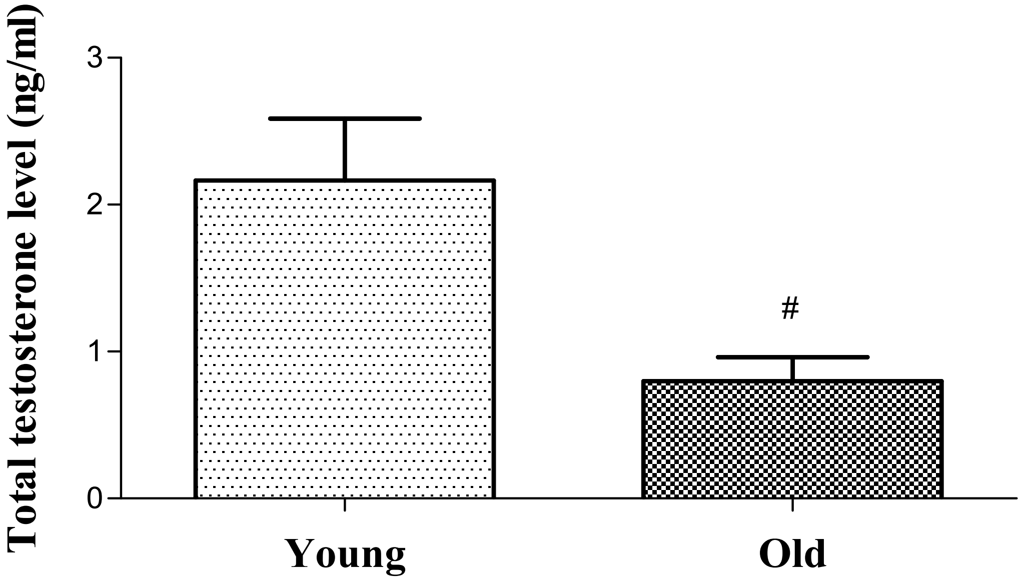

Figure S2. Comparative analysis of the total testosterone levels in young and old controls before KSB191 administration. Values are presented as mean ± SEM (n = 28). $^{\#}$ $p < 0.05$, compared with young controls. Statistical analyses were performed using the t-test.

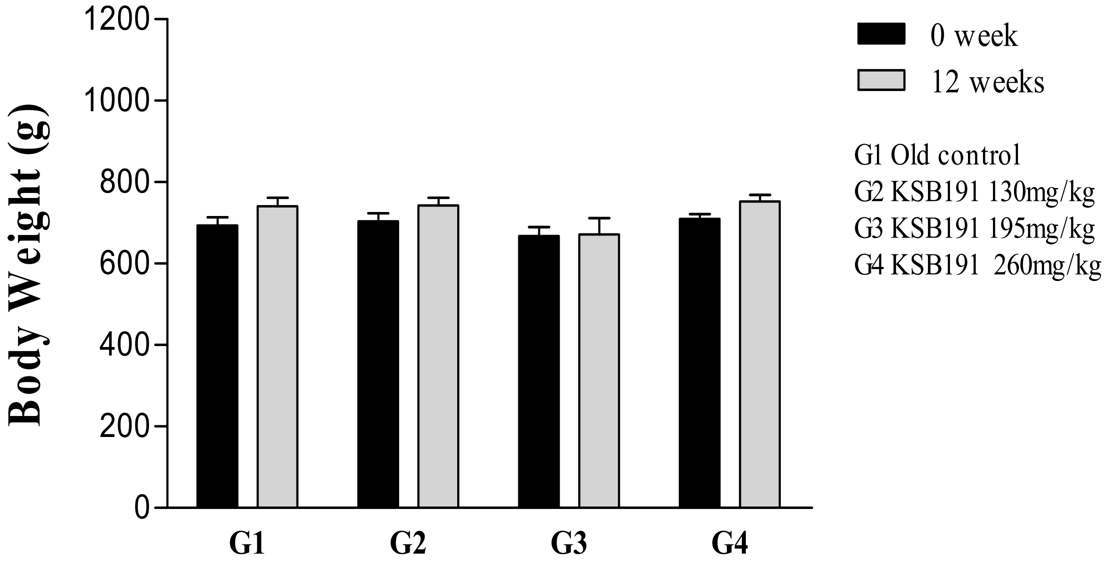

Figure S3. Comparative analysis of body weight before and after KSB191 administration for 12 weeks. Values are presented as mean ± SEM (n = 7). Statistical analyses were performed using t-test and ANOVA. G1, normal control; G2, 130 mg/kg KSB191; G3, 195 mg/kg KSB191; G4, 260 mg/kg KSB191

Table S1. Safety of KSB191 on organ weights

		**KSB191**		
	**G1. Old control**	**G2. 130 mg/kg**	**G3. 195 mg/kg**	**G4. 260 mg/kg**
**Thymus (g)**	0.092 ± 0.013	0.090 ± 0.014	0.085 ± 0.014	0.078 ± 0.013
**Kidney (g)**	4.052 ± 0.246	4.633 ± 0.523	3.648 ± 0.245	4.181 ± 0.365
**Liver (g)**	19.298 ± 1.118	19.486 ±0.799	18.276 ± 1.400	19.003 ± 1.265
**Testis (g)**	4.154 ± 0.110	4.173 ± 0.310	4.062 ± 0.081	4.394 ± 0.148
**Epididymis (g)**	1.495 ± 0.031	1.564 ± 0.103	1.518 ± 0.020	1.562 ± 0.046

Comparative analysis of organ weights at 12 weeks after KSB191 administration. Values are presented as mean ± SEM (n = 7). Statistical analyses were performed using t-test and ANOVA.

Table S2. Effect of KSB191 on lipid metabolism-related indicators

		KSB191		
	**G1. Old control**	**G2. 130 mg/kg**	**G3. 195 mg/kg**	**G4. 260 mg/kg**
**T-CHO (mg/dL)**	212.3 ± 25.1	180.9 ± 27.2	195.3 ± 27.8	166.4 ± 25.9
**TG (mg/dL)**	301.3 ± 55.9	258.3 ± 40.1	263.4 ± 78.9	242.1 ± 75.1

T-CHO, total cholesterol; TG, triglycerides. Values are presented as mean ± SEM (n = 7). Statistical analyses were performed using t-test and ANOVA.

Table S3. The sensitivity and the inter and intra variations of the ELISA kits

Hormones kit	sensitivity	Intra C.V.	Inter C.V.
Total testosterone (Abcam)	0.07 ng/mL	<= 5.8	<= 10.5
Free testosterone (MyBioSource)	1.0 pg/mL	<10%	<12%
Gonadotropin-releasing hormone (MyBioSource)	9.375 pg/ml	<8%	<10%
Luteinizing hormone (MyBioSource)	0.188mIU/ml	<8%	<10%
Follicle-stimulating hormone (MyBioSource)	1.88 ng/mL	<6.6%	<6.4%
